# Inverse relationship between ER-*β* and SRC-1 predicts outcome in endocrine-resistant breast cancer

**DOI:** 10.1038/sj.bjc.6602156

**Published:** 2004-10-12

**Authors:** E Myers, F J Fleming, T B Crotty, G Kelly, E W McDermott, N J O'Higgins, A D K Hill, L S Young

**Affiliations:** 1 Department of Surgery, Saint Vincent's University Hospital, Dublin 4, Ireland; 2 Department of Pathology, Saint Vincent's University Hospital, Dublin 4, Ireland; 3 Department of Statistics, University College Dublin, Dublin 4, Ireland; 4 Conway Institute, University College Dublin, Dublin 4, Ireland

**Keywords:** ER-*β*, SRC-1, breast cancer

## Abstract

The oestrogen receptor (ER) interacts with coactivator proteins to modulate genes central to breast tumour progression. Oestrogen receptor is encoded for by two genes, ER-*α* and ER-*β.* Although ER-*α* has been well characterized, the role of ER-*β* as a prognostic indicator remains unresolved. To determine isoform-specific expression of ER and coexpression with activator proteins, we examined the expression and localisation of ER-*α*, ER-*β* and the coactivator protein steroid receptor coactivator 1 (SRC-1) by immunohistochemistry and immunofluorescence in a cohort of human breast cancer patients (*n*=150). Relative levels of SRC-1 in primary breast cultures derived from patient tumours in the presence of *β*-oestradiol and tamoxifen was assessed using Western blotting (*n*=14). Oestrogen receptor-*β* protein expression was associated with disease-free survival (DFS) and inversely associated with the expression of HER2 (*P*=0.0008 and *P*<0.0001, respectively), whereas SRC-1 was negatively associated with DFS and positively correlated with HER2 (*P*<0.0001 and *P*<0.0001, respectively). Steroid receptor coactivator 1 protein expression was regulated in response to *β*-oestradiol or tamoxifen in 57% of the primary tumour cell cultures. Protein expression of ER-*β* and SRC-1 was inversely associated (*P*=0.0001). The association of ER-*β* protein expression with increased DFS and its inverse relationship with SRC-1 suggests a role for these proteins in predicting outcome in breast cancer.

In breast cancer, genomic actions of oestrogen are mediated through its receptor, leading to the transcription and translation of genes relevant to tumour progression. The oestrogen receptor (ER) is encoded by two genes, ER-*α* and ER-*β*, although the predominant form is ER-*α*, ER-*β* protein has been reported in 30–70% of breast cancers (Omoto *et al*, 2002; Fleming *et al*, 2004a; Palmieri *et al*, 2004). The role of ER-*β* in breast tumour progression, however, remains controversial. Differential signalling between ER-*α* and ER-*β* has been demonstrated with oestrogen and tamoxifen at the AP1 response element in ER target genes (Paech *et al*, 1997), suggesting that the ratio of ER-*α* to ER-*β* may result in alternate gene regulation and could consequently be important in determining the response to ER modulators, such as tamoxifen.

*Ex vivo* clinical studies undertaken to elucidate the prognostic significance of ER-*β* have, to date, been inconclusive. Early reports on ER-*β* mRNA expression in breast cancer described significant associations with axillary lymph node positivity and rate of tumour recurrence while on endocrine therapy (Speirs *et al*, 1999a, 1999b), lending credence to the hypothesis that ER-*β* is a poor prognostic indicator. Oestrogen receptor-*β* protein expression, however, has been associated with low tumour grade, negative axillary node status and a positive response to endocrine treatment (Jarvinen *et al*, 2000; Mann *et al*, 2001; Fleming *et al*, 2004a). Furthermore, ER-*β* protein expression was recently related to increased disease-free survival (DFS) and overall survival (Nakopoulou *et al*, 2004).

The extent of ER gene regulation is influenced not only by the ligand but also by the presence of specific coregulatory proteins, present at rate limiting levels, which modulate transcription. Over the past few years, a number of nuclear receptor interacting proteins have been isolated using various screening strategies. These include the p160 family of coactivator proteins, steroid receptor coactivator 1 (SRC-1) and amplified in breast cancer 1 (AIB1), both of which have intrinsic histone acelyl-transferase activity which enhance transcription by enabling access of transcription factors and RNA polymerase II core machinery to target DNA (Spencer *et al*, 1997; Liao *et al*, 2002). The relative expression of coregulatory proteins has been associated with tumour progression and the development of resistance to endocrine therapies. There is little consensus however as to the direction of these changes. Steroid receptor coactivator 1 and AIB1 mRNA levels have been associated with tumour progression; however, studies investigating coactivator RNA expression and resistance to ER modulators failed to demonstrate a significant association (Murphy *et al*, 2000, 2002). Recent studies have reported positive associations between AIB1 protein expression, high tumour grade and coexpression with the coactivators p300/CBP (Hudelist *et al*, 2003). We have previously described a positive relationship between SRC-1 expression and resistance to endocrine therapy (Fleming *et al*, 2004a) and have observed a significant association between both SRC-1 and AIB1 and the proto-oncogene HER2 (Fleming *et al*, 2004b).

We hypothesise that differences in expression and clinical correlations of ER isoforms in human breast cancer may be related to the altered response to the steroid environment and also the local expression of coactivator proteins. We have therefore examined the relationship between ER isoforms, ER-*α* and ER-*β*, and the coactivator protein SRC-1 in patients who developed disease recurrence.

## MATERIALS AND METHODS

### Patient population and clinical characteristics

A total of 150 breast carcinomas from patients who were diagnosed between 1996 and 2002 and six specimens of histologically normal breast tissue from patients undergoing reduction mammoplasties were examined. All patients had stage I–II breast cancer at presentation and were deemed free of distant metastases. All patients received chemotherapy and tamoxifen (20 mg day^−1^) for a maximum of 5 years. Tamoxifen was discontinued only in those patients who suffered a relapse while on endocrine therapy. In those patients who were ER negative, tamoxifen was prescribed on the basis of the fact that they were progesterone receptor positive. Those who received neoadjuvant chemo- or endocrine therapy were not included.

### Primary breast tumour cell cultures

Following ethical approval, breast tumour specimens were obtained from 14 patients undergoing surgery for removal of a histologically confirmed breast tumour. Approximately 1 cm^3^ of solid tumour was taken by the pathologist from the excision biopsy for cell culture, peripheral sections of which were removed and confirmed to be tumour by standard haematoxylin and eosin. Primary tumour epithelial cell cultures were prepared essentially as previously described (Fleming *et al*, 2004a). In brief, epithelial cells were extracted in HBSS without calcium or magnesium (Gibco, Paisley, Scotland) supplemented with 1 *μ*M EDTA and 1 *μ*M DTT for 40 min and cultured in RPMI containing 5 *μ*g ml^−1^ insulin, 10*μ*g ml^−1^ transferrin, 30 nM sodium selinate, 10 nM hydrocortisone, 10 nM *β*-oestradiol, 10 mM Hepes, 2 mM glutamine, 10% fetal calf serum (w v^−1^) and 5% ultroser G. To maximise cell attachment, cells were grown on a growth factor-reduced Matrigel matrix (BD Biosciences, San Jose, CA, USA) (60 ng cm^−2^). After 24 h, cells were washed in PBS and placed in phenol-free, serum and steroid-depleted MEM medium (Gibco) for 24 h before stimulation. Incubations were performed in the presence and absence of *β*-oestradiol and 4-hydroxytamoxifen (4-OHT) (10^−8^ M) for 24 h and harvested. Total protein was extracted using lysis buffer (1% Ipegal, 0.5% deoxycholic acid, 0.1% SDS and 1 × PBS) with pefabloc (5 *μ*g ml^−1^).

### Flow cytometry

Examination of primary breast cultures by staining with ethidium bromide and flow cytometric analysis using a phycoerythrin (PE)-labelled pan-leucocyte marker (CD45 RA and RO) confirmed cell viability and epithelial origin of tumour cells as previously described (Fleming *et al*, 2004a). Phenotypically distinct progenitor and nonprogenitor epithelial cell populations within the mammary epithelium were characterised. Flow cytometric analysis was carried out using a PE-conjugated monoclonal mouse anti-human EpCAM (epithelial specific antigen) antibody and FITC-conjugated mouse anti-human CD227 (MUC1) monoclonal mouse antibody (BD Biosciences). Biopotent progenitors (EpCAM^+^ MUC1^−^), which can generate both luminal and myoepithelial cells, were found to represent 51.9% of the epithelial specific antigen positive cell population, whereas the luminal restricted progenitor (EpCAM^+^ MUC^+^) were found to represent 48.1%.

### Immunohistochemistry

Thick tissue sections (5 *μ*m) were cut from paraffin-embedded breast tumour tissue and reduction mammoplasty blocks and mounted on Superfrost Plus slides (BDH, Poole, UK). Sections were dewaxed, rehydrated and washed in PBS. Endogenous peroxidase was blocked using 3% hydrogen peroxidase in PBS for 10 min. Antigen retrieval was performed by immersing sections in 0.6 M citrate buffer and microwaving on high power for 7 min. Immunohistochemistry was carried out using the Vectastain Elite kit (Vector Labs, Burlingame, CA, USA) according to the manufacturer's instructions. Briefly, sections were blocked in serum for 90 min. Sections were incubated with primary antibodies; rabbit anti-human ER-*α* (1 *μ*g ml^−1^), goat anti-human SRC-1 (1 *μ*g ml^−1^) (Santa Cruz, CA, USA) and mouse anti human ER-*β* (one in 20 in PBS) (Serotec, Oxford, UK) – for 60 min at room temperature. Antibodies for ER-*β* are directed against the wild-type ER-*β*, recognising the ER-*β*1 isoform of the protein. Sections were subsequently incubated with corresponding biotin-labelled secondary antibody (one in 2000) for 30 min, followed by peroxidase-labelled avidin–biotin complex. Sections were developed in 3,3-diaminobenzidine tetrahydrochloride (DAB) and counterstained with haematoxylin. Negative controls were performed using matched IgG controls (Dako, Denmark). Sections were examined under a light microscope. Immunostained slides were scored for ER-*α*, ER-*β* and SRC-1 using the Allred scoring system, as previously described (Harvey *et al*, 1999).

### Immunofluoresent microscopy

Breast cancer sections prepared as above were used for immunofluorescent studies. Slides were incubated in goat serum (ER-*α*) or sheep serum (ER-*β*) for 60 min. Rabbit anti-human ER-*α* (10 *μ*g ml^−1^ in 10% human serum) or mouse anti-human ER-*β* (1 : 2 dilution with PBS in 10% human serum) was placed on each slide for 90 min. The sections were rinsed in PBS and incubated with the corresponding secondary fluorochrome-conjugated antibody (one in 100) (Sigma-Aldrich, Steinheim, Germany) for 60 min. The slides were rinsed in PBS and blocked in rabbit serum for 90 min and subsequently washed again with PBS. Each slide was incubated with goat anti-human SRC-1 (10 *μ*g ml^−1^ with PBS in 10% human serum) for 90 min. The slides were incubated with the corresponding fluorochrome-conjugated antibody (one in 100) for 60 min. Sections were rinsed in PBS and mounted using fluorescent mounting media (DAKO, Denmark). Sections were examined under a fluorescent microscope. Negative controls were performed using matched IgG.

### Western blotting

Proteins (100 *μ*g) were resolved on a 12% polyacrylamide gel at 110 V for 120 min and then transferred to a nitrocellulose membrane (250 mA for 60 min). Membranes were incubated for 60 min in blocking buffer (5% nonfat dry milk, 0.1% Tween in PBS) at room temperature and subsequently with primary antibody, goat anti-rabbit SRC-1 (2 *μ*g ml^−1^), in blocking buffer overnight at 4°C. The membranes were washed prior to incubation with the corresponding secondary antibody (Santa Cruz Biotechnology Inc.) (one in 2000) in blocking buffer for 60 min at room temperature. The membranes were washed and developed with intensified luminescence (Pierce).

### Clinicopathological parameters

Variables analysed included patient age, tumour size, tumour grade, HER2 status and axillary node status. A recurrence was defined as any local (chest wall) or systemic (visceral or bone metastasis) recurrence during the follow-up period.

### Statistical analysis

Fisher's exact test was used in the comparison of two proportions throughout or equivalently to test for association in 2 × 2 tables. Kaplan–Meier estimates of survival functions were computed and the Wilcoxon test was used to compare survival curves. A Cox proportional hazards model was used to find significant predictors of disease-free survival (DFS). The predictors included in the model were: age, type of operation, axillary status, size of tumour, grade of tumour, AIBI, ER-*α*, ER-*β* and SRC-1. A stepwise procedure was used to find the best model. SAS statistical software (SAS Institute Inc., 1989) was used to perform these analyses.

## RESULTS

### Localisation of ER-*α*, ER-*β* and SRC-1 in human breast cancer

Oestrogen receptor-*α*, ER-*β* and the SRC-1 were localised in human breast tissue from patients with primary breast tumours and from patients undergoing normal reduction mammoplasties. Patient characteristics are given in Table 1

**Table 1:** Associations of ER-*α*, ER-*β* and SRC-1 with clinicopathological parameters, comparisons with Fishers exact test

	**Total**	**ER-*α* positivity (%)**	***P*-value**	**ER-*β* positivity (%)**	***P*-value**	**SRC-1 positivity (%)**	***P*-value**
No. of patients	150	123 (82%)	—	87 (58%)	—	28 (19%)	—
*Patient age (years)*							
⩽50	69	56 (81%)		35 (51%)		21 (30%)	
>50	81	67 (83%)	0.8338	52 (64%)	0.1009	7 (9%)	0.0007
							
*Tumour size (mm)*							
⩽35	107	85 (79%)		67 (63%)		9 (8%)	
>35	43	38 (88%)	0.2448	20 (47%)	0.0991	19 (44%)	<0.0001
							
*Histological grade*							
Grade 3	74	64 (86%)		46 (62%)		17 (23%)	
Non-Grade 3	76	59 (78%)	0.2030	41 (54%)	0.3256	11 (14%)	0.2120
							
*Node status*							
Node positive	94	78 (83%)		53 (56%)		20 (21%)	
Node negative	56	45 (80%)	0.8264	34 (61%)	0.6133	8 (14%)	0.3869
							
*HER-2-neu expression*							
Her-2-neu positivity	38	34 (89%)		10 (26%)		16 (42%)	
Her-2-neu negativity	112	89 (79%)	0.2234	77 (69%)	<0.0001	12 (11%)	<0.0001
							
*SRC-1 expression*							
SRC-1 positivity	28	25 (89%)		7 (25%)		—	—
SRC-1 negativity	122	98 (80%)	0.4130	80 (66%)	0.0001	—	
							
*Recurrences*							
Recurrence	29	26 (90%)		7 (24%)		26 (90%)	
Nonrecurrence	121	97 (80%)	0.2911	80 (66%)	<0.0001	2 (2%)	<.0001

. The median follow-up was 27 months.

**Figure 1:**
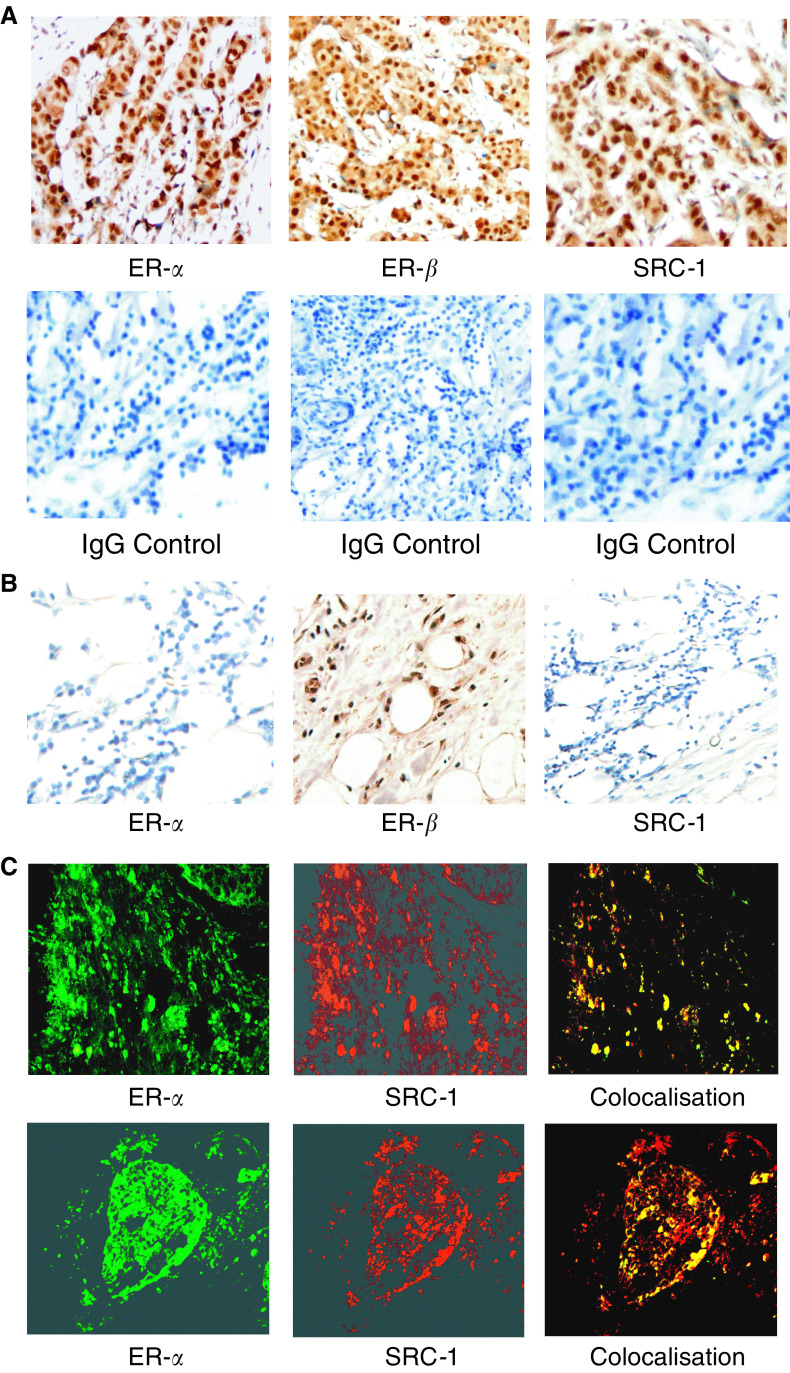
ER-*α*, ER-*β* and SRC-1 protein expression in paraffin-embedded invasive breast carcinoma and normal breast tissue specimens. (**A**) Immunohistochemical localisation of ER-*α*, ER-*β* and SRC-1 (× 200) in primary breast cancer counterstained with haematoxylin and matched IgG controls. (**B**) ER-*α*, ER-*β* and SRC-1, (× 200) protein expression in normal breast tissue. (**C**) Immunofluorescent colocalisation of ER-*α* with SRC-1 (× 200) and ER-*β* with SRC-1 (× 200) invasive breast carcinoma.

Both ER isoforms were localised to the nuclei of primary breast tumour epithelial cells (Figure 1A

). However, while ER-*α* was expressed almost exclusively in the nuclei of tumour cells, distinct cytoplasmic expression of ER-*β* was observed. Oestrogen receptor-*β*, but not ER-*α*, was found to be expressed in normal tissue surrounding the tumour and in tissue sections from normal reduction mammoplasties. Steroid receptor coactivator 1 was localised to the nuclei of breast tumour epithelial cells, but was not observed in normal breast tissue (Figure 1B). Colocalisation of both ER-*α* and ER-*β* with SRC-1 in breast tumour epithelial cells was confirmed using immunofluorescence (Figure 1C).

ER-*α* and ER-*β* were found to be expressed in 82 and 58% of breast tumour patients, respectively, whereas SRC-1 was detected in 19% of breast tumours (Table 1). Oestrogen receptor-*α* and ER-*β* were coexpressed in 46.6% of tumours. Oestrogen receptor-*α* and SRC-1 were coexpressed in 17.3%, whereas ER-*β* and SRC-1 were both expressed in 4.6% of breast tumours. There was no significant correlation observed between ER-*α* protein expression and SRC-1 (*P*=0.4130), however, ER-*β* was found to be inversely associated with the steroid receptor coactivator (*P*=0.0001).

### Correlation of ER-*α*, ER-*β* and SRC-1 with clinical variables

Associations between the qualitative expression of ER-*α*, ER-*β* and SRC-1 and clinicopathological parameters were examined. No relationship between the expression of the ER isoforms was observed in relation to patient age, tumour size, histological grade and axillary node status (Table 1). Steroid receptor coactivator 1 was found to positively associate with tumour size (*P*<0.0001), and HER2 status (*P*<0.0001), whereas ER-*β* inversely associated with HER2 expression (*P*<0.0001) (Table 1).

**Figure 2:**
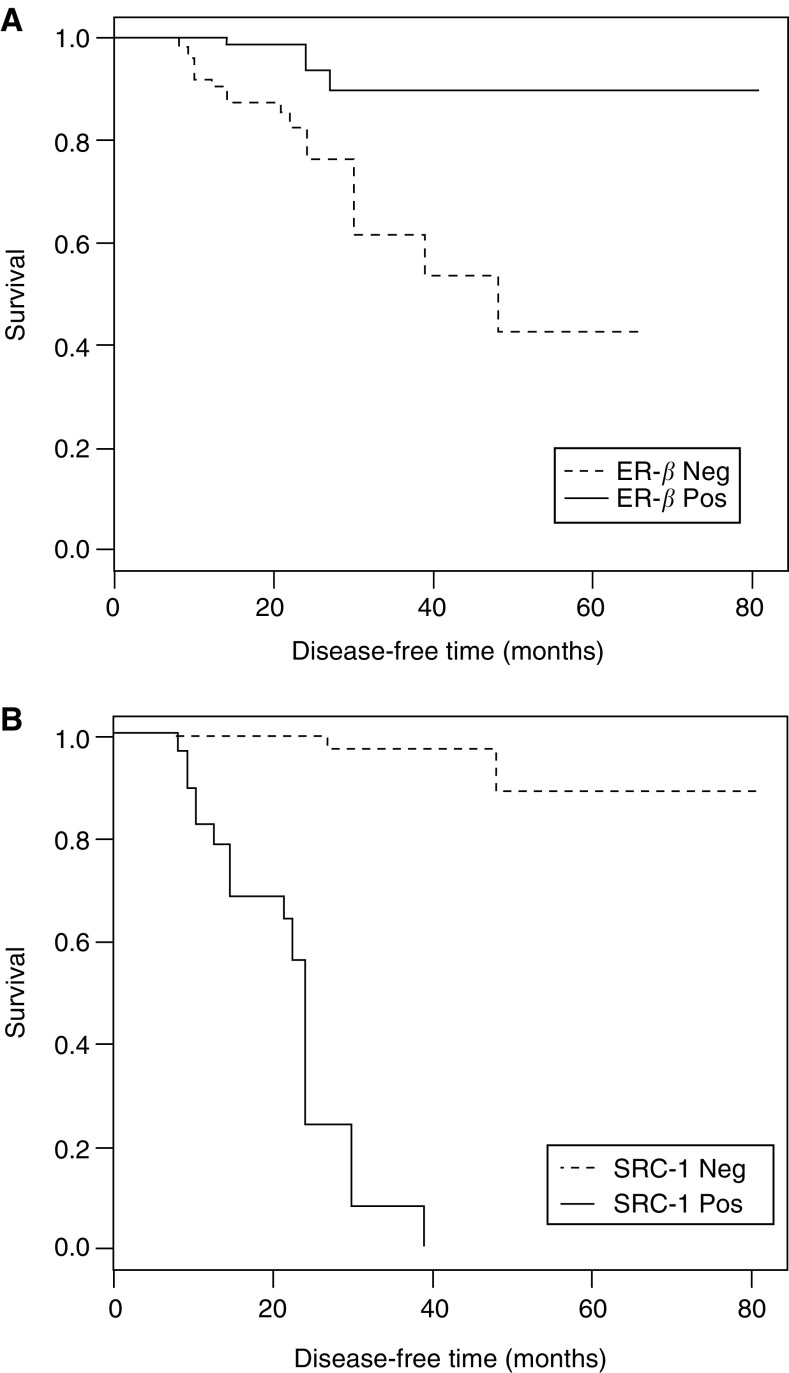
Kaplan–Meier estimates of disease-free survival (DFS). (**A**) Disease-free survival according to ER-*β* expression and (**B**) according to SRC-1 expression.

From Kaplan–Meier estimates of survival, ER-*β* protein expression was found to be significantly associated with DFS (*P*=0.0008), conversely SRC-1 significantly associated with disease recurrence (*P*<0.0001), (Figure 2

). Using a Cox proportional hazards model, significant predictors of DFS were identified. The final model included SRC-1 and axillary node status as significant predictors (*P*<0.0001 and *P*=0.0193, respectively). Although ER-*β* was also a significant predictor of DFS, it is not required in the model in the presence of SRC-1, due to the strong association between ER-*β* and SRC-1 expressions.

### Response to endocrine environment

**Figure 3:**
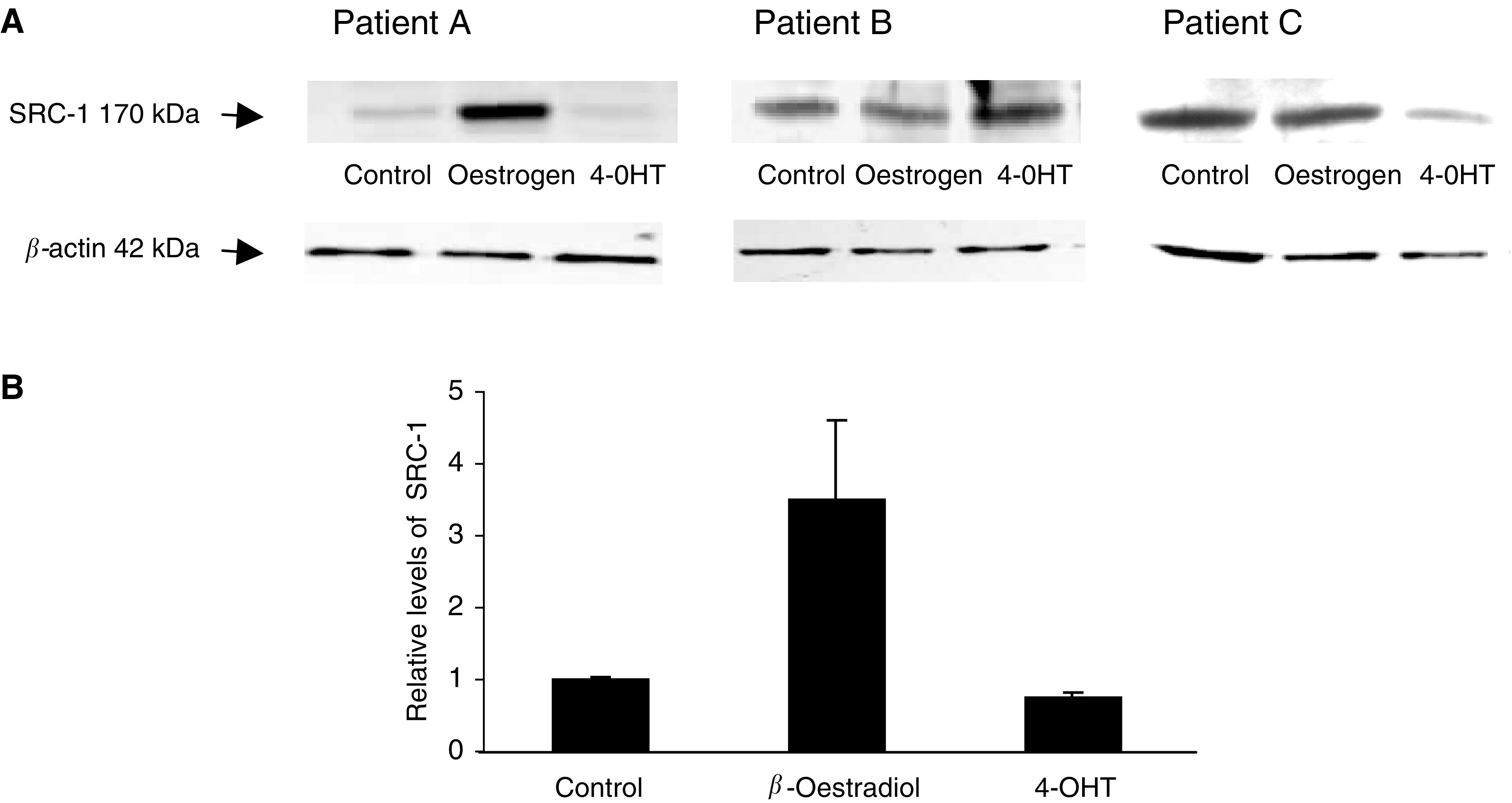
Western blot analysis of SRC-1 protein levels in primary breast cultures. (**A**) Illustrative blots of primary breast tumour response to steroid environment; patient A – increased expression of SRC-1 in response to oestrogen, patient B – nonresponder to oestrogen or tamoxifen, patient C – decreased expression of SRC-1 in response to tamoxifen. (**B**) Relative optical density of SRC-1 immunoblots. Optical density readings of control values were normalised to 1 and treated groups were expressed as a ratio. Values are expressed as mean±s.e.m. (*n*=14).

The ability of SRC-1 protein to be regulated by oestrogen and the selective ER modulator, tamoxifen (4-OHT), was examined in primary breast cell cultures derived from patient tumours, median follow-up 4 months (*n*=14) (Figure 3

). Basal levels of SRC-1 were detected in all cultures. Treatment with oestrogen induced an increase in SRC-1 protein expression, compared to basal levels, in 28% of tumour cell cultures, whereas tamoxifen induced a reduction in SRC-1 expression in a similar number of breast cancer patients (Table 2

**Table 2:** Response of SRC-1 to steroid environment in primary breast tumour cell cultures

**Patient**	**Age**	**Lymph node status**	**Grade**	**ER**	**PR**	**HER2**	**SRC-1+*β*-oestradiol**	**SRC-1 +4-OHT**
1	60	P	2	P	P	N	—	↓
2	75	P	3	N	N	P	↑	—
3	76	P	2	P	N	N	—	—
4	55	P	3	P	N	P	↑	—
5	70	P	3	P	P	N	—	↓
6	46	P	2	P	P	N	—	—
7	50	N	2	N	P	N	—	—
8	48	N	3	P	P	N	—	—
9	41	N	2	P	N	N	—	—
10	39	P	3	N	P	P	—	—
11	45	P	3	P	P	P	↑	—
12	71	P	2	P	N	N	—	↓
13	49	N	2	P	N	N	—	↓
14	32	P	3	P	P	P	↑	—

). Of interest, one patient which was ER negative increased protein expression of SRC-1 in response to treatment with *β*-oestradiol.

The relative response to oestrogen and tamoxifen treatment was examined in relation to the patient clinical characteristics. Of interest, tumours that upregulated SRC-1 protein expression in response to oestrogen were also found to be HER2 positive and those that reduced SRC-1 expression in the presence of tamoxifen were HER2 negative (Table 2).

## DISCUSSION

The prognostic significance of ER-*β* protein expression in human breast cancer remains controversial. Altered response to oestrogen and ER modulators between ER-*α* and ER-*β* in relation to receptor DNA binding, activation of target genes and *in vivo* tumour formation has been described (Paech *et al*, 1997; Paruthiyil *et al*, 2004), leading to the possibility that the two ER isoforms may have distinct roles in disease progression. *Ex vivo* studies examining the expression of ER-*β* mRNA in relation to clinicopathological parameters have described associations between ER-*α* and ER-*β* coexpression and axillary node positivity (Speirs *et al*, 1999a), and in a limited series, between ER-*β* mRNA and resistance to endocrine treatment (Speirs *et al*, 1999b). However, at the protein level using antibodies directed against the NH2-terminal region of ER-*β*, detecting both full-length ER-*β* (ER-*β*1) and various COOH-terminal truncated isoforms, Fuqua *et al* (2003) found no correlation between ER-*β* expression and tumour grade, proliferation, S-phase fraction or DNA ploidy, while others found ER-*β* status to be a significant predictor of response to endocrine therapy (Mann *et al*, 2001; Fleming *et al*, 2004a). Furthermore, reductions in ER-*β* protein expression have been associated with the development of an invasive phenotype (Skliris *et al*, 2003). These studies raise the possibility that ER-*β* may function as a tumour suppressor and that loss of ER-*β* could promote tumourogenesis. In this study using antibodies directed against the COOH-terminal, we examined the expression of wild-type ER-*β* (ER-*β*1) in relation to established clinical parameters of breast cancer. We found a positive association between ER-*β* protein expression and DFS, furthermore, a significant inverse relationship between ER-*β* and the proto-oncogene HER2 was also observed.

A role for nuclear coregulatory proteins in ER-mediated transactivation of target genes has been established. We and others have described altered recruitment of coactivator proteins to the ER in the presence of oestrogen and ER modulators (Bai and Giguere, 2000; Margeat *et al*, 2003; Fleming *et al*, 2004a). We have previously observed that SRC-1 protein expression was associated with resistance to endocrine therapy (Fleming *et al*, 2004a) and the growth factor receptor HER2 (Fleming *et al*, 2004b). Here, we describe a significant inverse association between SRC-1 and DFS. When we related ER-*β* and SRC-1 protein expression, we found a significant inverse relationship, suggesting that the role of ER-*β* as a good prognostic indicator may be due, at least in part, to low expression levels of the p160 coactivator SRC-1. We examined the ability of oestrogen and the ER modulator, tamoxifen, to regulate SRC-1 protein expression in primary breast cell cultures derived from patient tumours and found that approximately 28% of cultures increased protein expression of SRC-1 in response to oestrogen, whereas the same percentage, but different patient group, reduced SRC-1 in the presence of tamoxifen. Of interest one of the tumours that upregulated SRC-1 protein expression in response to *β*-oestradiol was ER negative. This may reflect oestrogen functioning in a nongenomic fashion, independently of the nuclear receptor ER. Oestrogen actions in ER-negative breast cell lines have previously been described (Filardo *et al*, 2000).

When response to endocrine environment was related to clinical characteristics, we found that those tumours which upregulated SRC-1 in the presence of oestrogen were all HER2-positive tumours and conversely those that could downregulate SRC-1 in response to tamoxifen were HER2 negative. The relationship between SRC-1 response to the steroid environment and HER2 status is an interesting one, as p160 proteins are known to be phosphorylated via the MAP kinase pathway (Font de Mora and Brown, 2000; Rowan *et al*, 2000). Taken together, these data provide further evidence of the proposed crosstalk between growth factor receptor pathways and activation of steroid receptor coactivator proteins (Johnston *et al*, 2003; Osborne *et al*, 2003).

The observation that SRC-1 expression is strongly associated with the development of recurrence and a reduced DFS requires validation in a large cohort of breast cancer patients to establish its potential as a meaningful prognostic marker in breast cancer. The association of ER-*β* protein expression with increased DFS and its inverse relationship with the coregulatory protein SRC-1 suggests a significant role for these proteins in determining outcome in breast cancer.
